# Evaluation of end-tidal carbon dioxide gradient as a predictor of volume responsiveness in spontaneously breathing healthy adults

**DOI:** 10.1186/s40635-018-0187-0

**Published:** 2018-07-30

**Authors:** María C. Arango-Granados, Virginia Zarama Córdoba, Andrés M. Castro Llanos, Luis A. Bustamante Cristancho

**Affiliations:** 1 Universidad Icesi, Calle 18 No. 122-135 Pance, Cali, Colombia; 2 Fundación Valle del Lili, Av. Simón Bolívar. Cra 98 # 18-49, Cali, Colombia

**Keywords:** Capnography, Cardiac output, Doppler echocardiography, Blood volume determination, Hemodynamic monitoring

## Abstract

**Background:**

Methods to guide fluid therapy in spontaneously breathing patients are scarce. No studies have reported the accuracy of end-tidal CO_2_ (ET-CO_2_) to predict volume responsiveness in these patients. We sought to evaluate the ET-CO_2_ gradient (ΔET-CO_2_) after a passive leg rise (PLR) maneuver to predict volume responsiveness in spontaneously breathing healthy adults.

**Methods:**

We conducted a prospective study in healthy adult human volunteers. A PLR maneuver was performed and cardiac output (CO) was measured by transthoracic echocardiography. ET-CO2 was measured with non-invasive capnographs. Volume responsiveness was defined as an increase in cardiac output (CO) > 12% at 90 s after PLR.

**Results:**

Of the 50 volunteers, 32% were classified as volume responders. In this group, the left ventricle outflow tract velocity time integral (VTI_LVOT_) increased from 17.9 ± 3.0 to 20.4 ± 3.4 (*p* = 0.0004), CO increased from 4.4 ± 1.5 to 5.5 ± 1.6 (*p* = 0.0), and ET-CO_2_ rose from 32 ± 4.84 to 33 ± 5.07 (*p* = 0.135). Within the entire population, PLR-induced percentage ∆CO was not correlated with percentage ∆ET-CO_2_ (*R*^2^ = 0.13; *p* = 0.36). The area under the receiver operating curve for the ability of ET-CO_2_ to discriminate responders from non-responders was of 0.67 ± 0.09 (95% CI 0.498–0.853). A ΔET-CO_2_ ≥ 2 mmHg had a sensitivity of 50%, specificity of 97.06%, positive likelihood ratio of 17.00, negative likelihood ratio of 0.51, positive predictive value of 88.9%, and negative predictive value of 80.5% for the prediction of fluid responsiveness.

**Conclusions:**

ΔET-CO_2_ after a PLR has limited utility to discriminate responders from non-responders among healthy spontaneously breathing adults.

**Electronic supplementary material:**

The online version of this article (10.1186/s40635-018-0187-0) contains supplementary material, which is available to authorized users.

## Background

One of the most important goals during the resuscitation of the critically ill patient is to reestablish an adequate oxygen delivery (DO2) to the tissues. Given the theory that DO2 depends, among others, on cardiac output (CO), the clinician may consider to give intravenous fluids aiming to increase left ventricular preload and stroke volume (SV). However, it is known that not every patient responds to a volume challenge with an increase in CO. Furthermore, fluid administration requires careful monitoring because both, volume deficit and overload, can worsen the prognosis [1, 2].

For this reason, the fundamental challenge is to accurately estimate if the patient will benefit from a volume challenge. Methods to guide fluid therapy in the spontaneously breathing patient are scarce, and many of them require invasive monitoring [3]. This may limit its routine use in patients who present to the emergency department or who are being treated in scenarios that do not have these monitoring tools within reach.

An interesting and non-invasive tool is the evaluation of the aortic flow by transthoracic echocardiography (TTE). Variables such as the change in peak aortic flow velocity and left ventricle outflow tract velocity time integral (VTI_LVOT_) after a passive leg rise (PLR) maneuver are good predictors of volume responsiveness in critically ill patients (sensitivity 77% and 100% specificity) [4, 5]; however, this echocardiographic measurements require more advanced training, can be difficult to estimate in patients with inadequate acoustic windows, there are important limitations with keeping the same angle between probe and the left outflow tract in both positions, and finally, these measurements take a considerable time to obtain.

The relationship between expired CO_2_ (ET-CO_2_) and CO has been known for several decades [6, 7]. Since ET-CO_2_ is determined mainly by the tissue production of carbon dioxide (CO_2_), alveolar ventilation and CO [8], under constant metabolic and ventilatory conditions, acute changes in ET-CO_2_ have shown to correlate strongly with changes in CO in experimental [9–11] and clinical [12, 13] scenarios. For this reason, the variation in ET-CO_2_ has been used as a method to predict volume responsiveness after a PLR maneuver in the mechanically ventilated patient [14].

To date, there are no published studies evaluating the accuracy of non-invasive ET-CO_2_ to predict volume responsiveness in the spontaneously breathing patient. It is well known that healthy individuals can work at various points of the Frank-Starling curve at different times due to small changes in their cardiac contractility and/or effective vascular volume, so a percentage of them can respond to volume at a given moment [15]. Therefore, the objective of this study is to evaluate the performance of the expired CO_2_ gradient (ΔET-CO_2_) after a PLR maneuver to predict volume responsiveness in spontaneously breathing healthy adults.

## Methods

### Study population

We included human volunteers of both sexes, ≥ 18 years, classified according to the American Society of Anesthesiologists as ASA I or ASA II. This classification (ASA I and II) includes healthy people or with mild systemic diseases without major functional limitations [16]. Volunteers who presented arrhythmias at the time of analysis, lower limb amputation, inadequate cardiac window for VTI_LVOT_ measurement, pregnant women, participants who during the maneuver did not tolerate supine position or leg rise, and finally, patients who refused to participate were excluded.

A non-probability convenience sample was used, estimating a total of at least 50 participants. Volunteers were mainly medical school students, residents, hospital staff, and close contacts of the researchers (family, friends, and colleagues). This study was conducted in the Valle del Lili Foundation Hospital (Cali, Colombia) after approval by the Institutional Ethics Committee. All informed consents are duly signed and stored in this department.

### Measurements

Participants were contacted individually and required to be fasted, according to the definition of fasting of the American Society of Anesthesiology [17]. In the laboratory, after 2 min of placing the participants on a supine position with head at 45°, baseline hemodynamic variables were recorded (Additional file 1: Table S1). Subsequently, the PLR maneuver was performed, according to the original description of the technique: semi recumbent position, total supine position with leg rise at 45 ° for 90 s, return to the basal position [18]. The different hemodynamic variables, including capnography, were recorded at 30, 60, 90, 5, 8, and 10 min after the initiation of the maneuver. VTI_LVOT_ measurement was taken 90 s, 5 min, and 10 min after the PLR.

For the purpose of this study, the participant who had an increase in CO > 12% after 90 s of PLR was classified as a volume responder. CO was calculated by measuring the left ventricular outflow tract diameter (in the parasternal long axis view), the VTI_LVOT_ (in the apical five-chamber view), and the heart rate (CO=$\pi {\left(\frac{D}{2}\right)}^2\times \mathrm{VTI}\times \mathrm{HR}\Big)$. All echocardiographic evaluations were performed by experienced sonographers (VZ, LB, CV).

Capnography was measured using Nihon Khoden cap-ONE® TG-920P mainstream CO_2_ sensor, attached to a disposable oral and nasal adaptor and placed directly at the point of expiration. The other hemodynamic variables and their source are described in Additional file 1: Table S1.

### Data management and statistical analysis

An information quality control to determine missing data and extreme data was carried out. A Shapiro-Wilk test was used to determine the distribution of the numerical variables; these were summarized as mean and standard deviations or median and interquartile ranges, as appropriate. Categorical variables were summarized as proportions.

Differences between responders and non-responders were compared by means of an independent sample *t* test, except for age, SpO2, VTI_LVOT_, SV, CO, and cardiac index, which were compared by the Mann-Whitney *U* test.

The effects of PLR on hemodynamic parameters were assessed using a paired Student’s *t* test. The area under the receiver operating curve (ROC) was calculated using the Hanley-McNeil test. ROC curves are presented as area ± SE (95% confidence interval). ET-CO_2_’s capability to track changes in CO during PLR was tested using a concordance analysis through a Pearson correlation coefficient, both for percentage changes and for absolute values at each measuring point.

The best cutoff point was identified and reported in terms of sensitivity, specificity, positive predictive value (PPV), negative predictive value (NPV), positive likelihood ratio (LR+), and negative likelihood ratio (LR−). For this purpose, individuals with a decrease in ET-CO_2_ after PLR were assumed to have an increase of 0 mmHg. Trend graphs were constructed to evaluate the behavior of the hemodynamic variables over time (30 s–10 min). All statistical analyses were performed using STATA software. A *p* value < 0.05 was considered statistically significant.

## Results

**Fig. 1:**
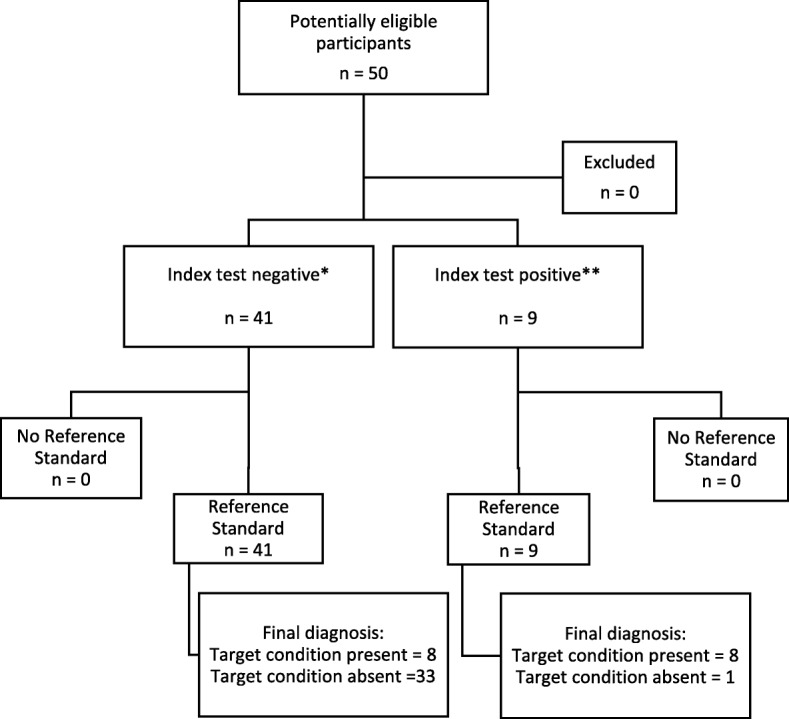
Flow of participants through the study. *Defined as ∆ET-CO2 < 2 mmHg; **defined as ∆ET-CO2 ≥ 2 mmHg

A total of 50 volunteers were included in the final analysis. The flow of participants through the study is shown in Fig. 1. A large proportion of the population (82%) had no past medical history; only 2 of them were hypertensive, 1 hypothyroid, 1 with sinus bradycardia, 2 with premature ventricular contractions, 2 with mild mitral regurgitation, 1 with a history of vasovagal syncope, and 1 with a history of stage 2 chronic kidney disease. The mean baseline VTI_LVOT_ was 18.8 ± 2.8 cm, baseline CO was 4.3 ± 1.1 L/min, and baseline ET-CO_2_ was 32.0 ± 3.5 mmHg (Table 1).

**Table 1:** Characteristics of the study population

	Total, *n* = 50	Non-responders, *n* = 34	Responders, *n* = 16	*p* value^#^
Age (years)^β, Ω^	27 (23–30)	26 (23–29)	30 (23–35.5)	0.110
Male, *n* (%)	26 (52)	17 (50)	9 (56)	0.68
Latin American, *n* (%)	47 (94)	31 (91.2)	16 (100)	0.542
Afrodescendant, *n* (%)	3 (6)	1 (2.9)	0 (0)	–
Weight (kg), mean ± SD	71.48 ± 15.99	68.29 ± 15.39	78.25 ± 15.56	0.039
Height (m), mean ± SD	1.68 ± 3.6	1.67 ± 0.11	1.71 ± 0.11	0.293
Body mass index (kg/m^2^), mean ± SD	24.98 ± 3.60	24.18 ± 3.37	26.72 ± 3.58	0.019
Body surface area (m^2^), mean ± SD	1.82 ± 0.25	1.77 ± 0.25	1.92 ± 0.24	0.058
Heart rate (bpm), mean ± SD	73 ± 12.55	73 ± 13.01	74 ± 11.92	0.853
Systolic blood pressure (mmHg), mean ± SD	118 ± 12.74	113 ± 11.96*	128 ± 8.87	0.0001
Diastolic blood pressure (mmHg), mean ± SD	73 ± 8.94	71 ± 9.21*	77 ± 6.83	0.011
Mean arterial pressure (mmHg), mean ± SD	89 ± 11.53	86 ± 10.67**	96 ± 10.28	0.002
Peripheral capillary oxygen saturation (%)^β, Ω^	98 (98–99)	98 (98–99)	97 (96.5–98)	0.051
Respiratory rate (rpm), mean ± SD	17 ± 4.71	17 ± 4.88	17 ± 4.5	0.845
End-tidal carbon dioxide (mmHg), mean ± SD	32 ± 3.50	32 ± 2.76	32 ± 4.84	0.911
End-tidal carbon dioxide (mmHg/respiratory rate^β, Ω^	1.83 (1.5–2.46)	1.81 (1.52–2.5)	1.96 (1.46–2.17)	0.925
Left ventricle outflow tract VTI (cm)^β, Ω^	18.2 (17.3–21)	18.9 (17.97–21.1)	17.9 (16.45–18.2)	0.075
Left ventricle outflow tract area (cm^2^), mean ± SD	3.2 ± 0.68	3.1 ± 0.65	3.4 ± 0.76	0.191
Stroke volume (mL), mean ± SD	60.1 ± 16.41	60.0 ± 16.07	60.5 ± 17.66	0.924
Stroke volume index (mL/m^2^)^β, Ω^	31.3 (25.34–37.51)	30.7 (25.2–38.58)	32.8 (28.41–37.36)	0.406
Cardiac output (L/min)^β, Ω^	4.2 (3.53–4.92)	4.3 (3.54–4.92)	4.2 (3.31–4.91)	0.901
Cardiac index (L/min/m^2^)^β, Ω^	2.3 (1.89–2.79)	2.1 (1.87–2.79)	2.4 (2.08–2.8)	0.34

*SD* standard deviation, *bpm* beats per minute, *rpm* respirations per minute, *VTI* velocity time integral

^#^Comparison between responders and non-responders

^β^Median (interquartile range)

^Ω^Mann-Whitney test

*n = 32

**n = 31

**Fig. 2:**
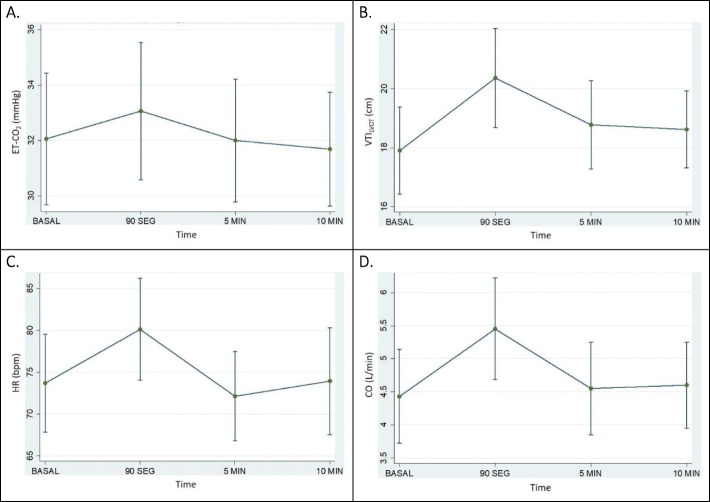
Among responders, behavior during PLR maneuver of **a** ET-CO2, **b** VTI_LVOT_, **c** HR, and **d** CO. ET-CO2, end-tidal carbon dioxide; VTI_LVOT_, left ventricle outflow tract velocity time integral; HR, heat rate; CO cardiac output

**Fig. 3:**
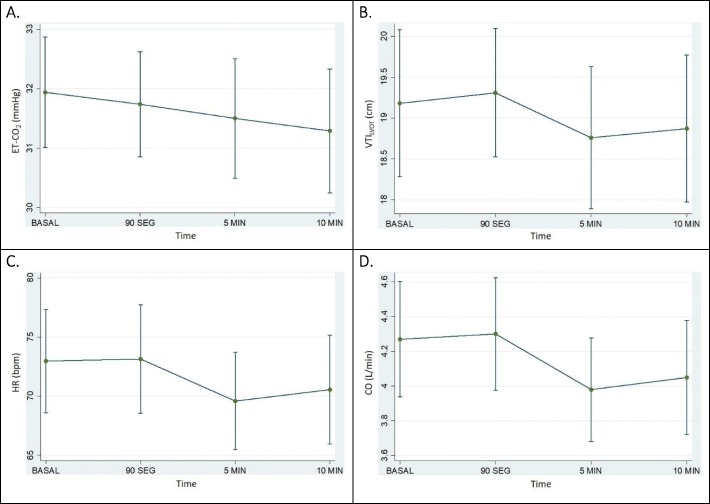
Among non-responders, behavior during PLR maneuver of **a** ET-CO2, **b** VTI_LVOT_, **c** HR, and **d** CO. ET-CO2, end-tidal carbon dioxide; VTI_LVOT_, left ventricle outflow tract velocity time integral; HR, heat rate; CO, cardiac output

Of the 50 volunteers, a total of 16 (32%) were classified as volume responders after the PLR. In this group of participants, the VTI_LVOT_ increased from 17.9 ± 3.01 to 20.4 ± 3.42 (*p* = 0.0004), CO increased from 4.4 ± 1.45 to 5.5 ± 1.57 (*p* = 0.00), and ET-CO_2_ rose from 32 ± 4.84 to 33 ± 5.07 (*p* = 0.135) (Table 2). In the group of non-responders, the VTI_LVOT_ did not show significant changes (from 19.2 ± 2.67 to 19.3 ± 2.33, *p* = 0.636), nor did the CO (4.3 ± 0.99 to 4.3 ± 0.96, *p* = 0.474) or the ET-CO_2_ (32 ± 2.76 to 32 ± 2.63, *p* = 0.408) (Table 3). Changes in ET-CO_2_, VTI_LVOT_, HR, and CO throughout the PLR maneuver among responders and non-responders are shown in Figs. 2 and 3.

**Table 2:** Hemodynamic parameters pre- and post-PLR among responders

	Basal	90 s	*p* value^#^	5 min	*p* value^β^	10 min	*p* value^Ω^
Heart rate (bpm)*	74 ± 11.92	80 ± 12.45	0.003	72 ± 10.89	0.210	74 ± 12.99	0.892
Systolic blood pressure (mmHg)*	128 ± 8.87	122 ± 12.64	0.076	122 ± 11.62	0.050	118 ± 11.44	0.013
Diastolic blood pressure (mmHg)*	77 ± 6.83	72 ± 8.86	0.081	72 ± 8.94	0.039	74 ± 9.32	0.214
Mean arterial pressure (mmHg)*	96 ± 10.28	88 ± 11.62	0.049	90 ± 11.58	0.060	90 ± 11.47	0.072
Peripheral capillary oxygen saturation (%)*	97 ± 1.2	97 ± 1.54	0.530	97 ± 1.26	0.485	97 ± 1.48	0.138
Respiratory rate (rpm)*	17 ± 4.5	16 ± 5.07	0.368	16 ± 4.31	0.166	16 ± 4.95	0.169
ET-CO2 (mmHg)*	32 ± 4.84	33 ± 5.07	0.135	32 ± 4.52	0.917	32 ± 4.19	0.478
ET-CO2/RR*	1.98 ± 0.63	2.35 ± 1.19	0.187	2.16 ± 0.75	0.178	2.25 ± 0.96	0.156
Left ventricle outflow tract VTI (cm)*	17.9 ± 3.01	20.4 ± 3.42	0.0004	18.8 ± 3.06	0.139	18.6 ± 2.66	0.162
Left ventricle outflow tract area (cm^2^)*	3.4 ± 0.76		NA		NA		NA
Stroke volume (mL)*	60.5 ± 17.66	68.9 ± 19.36	0.0009	63.3 ± 16.95	0.111	63.3 ± 18.21	0.098
Stroke volume index (mL/m^2^)*	35.5 ± 11.42	40.3 ± 12.05	0.0006	37.1 ± 11.34	0.101	37.1 ± 11.6	0.115
Cardiac output (L/min)*	4.4 ± 1.45	5.5 ± 1.57	0	4.6 ± 1.43	0.388	4.6 ± 1.32	0.259
Cardiac index (L/min/m^2^)*	2.6 ± 0.88	3.2 ± 0.94	0	2.7 ± 0.89	0.356	2.7 ± 0.83	0.281

*SD* standard deviation, *bpm* beats per minute, *rpm* respirations per minute, *ET-CO2* end-tidal carbon dioxide, *RR* respiratory rate, *VTI* velocity time integral, *NA* does not apply

#Comparison of 90 s against baseline parameters

^β^Comparison of 5 min against baseline parameters

^Ω^Comparison of 10 min against baseline parameters

*Paired *t* test

**Table 3:** Hemodynamic parameters pre- and post-PLR among non-responders

	Basal	90 s	*p* value^#^	5 min	*p* value^β^	10 min	*p* value^Ω^
Heart rate (bpm)*	73 ± 13.01	73 ± 13.65	0.848	70 ± 12.23	0	71 ± 13.74	0.007
Systolic blood pressure (mmHg)*, *n* = 32	113 ± 11.96	112 ± 12.03	0.468	111 ± 11.39	0.201	110 ± 10.90	0.091
Diastolic blood pressure (mmHg)*, *n* = 32	71 ± 9.21	67 ± 9.56	0.047	67 ± 8.08	0.036	67 ± 8.37	0.008
Mean arterial pressure (mmHg)*, *n* = 31	86 ± 10.67	83 ± 10.41	0.183	83 ± 9.09	0.138	81 ± 9.75	0.025
Peripheral capillary oxygen saturation (%)*	98 ± 1.21	98 ± 1.32	0.786	98 ± 1.85	0.088	98 ± 1.64	0.231
Respiratory rate (bpm)*	17 ± 4.88	18 ± 5.98	0.113	17 ± 4.4	0.939	16 ± 4.31	0.484
ET-CO2 (mmHg)*	32 ± 2.76	32 ± 2.63	0.408	32 ± 3	0.092	31 ± 3.1	0.019
ET-CO2/RR*	2.08 ± 0.82	2.03 ± 1.11	0.784	2.02 ± 0.77	0.597	2.11 ± 0.85	0.892
Left ventricle outflow tract VTI (cm)*	19.2 ± 2.67	19.3 ± 2.33	0.636	18.8 ± 2.58	0.111	18.9 ± 2.67	0.239
Left ventricle outflow tract area (cm^2^)*	3.1 ± 0.65		NA		NA		NA
Stroke volume (mL)*	60.0 ± 16.07	60.6 ± 16.33	0.552	58.6 ± 15.73	0.101	58.9 ± 15.6	0.195
Stroke volume index (mL/m^2^)*	32.6 ± 10.17	33.1 ± 10.22	0.375	32.1 ± 10.36	0.321	32.2 ± 9.88	0.371
Cardiac output (L/min)*	4.3 ± 0.99	4.3 ± 0.96	0.474	4.0 ± 0.89	0	4.0 ± 0.98	0.014
Cardiac index (L/min/m^2^)*	2.3 ± 0.6	2.3 ± 0.58	0.309	2.2 ± 0.59	0.0008	2.2 ± 0.6	0.036

*SD* standard deviation, *bpm* beats per minute, *rpm* respirations per minute, *ET-CO2* end-tidal carbon dioxide, *RR* respiratory rate, *VTI* velocity time integral, *NA* does not apply

^#^Comparison of 90 s against baseline parameters

^β^Comparison of 5 min against baseline parameters

^Ω^Comparison of 10 min against baseline parameters

*Paired *t* test

**Fig. 4:**
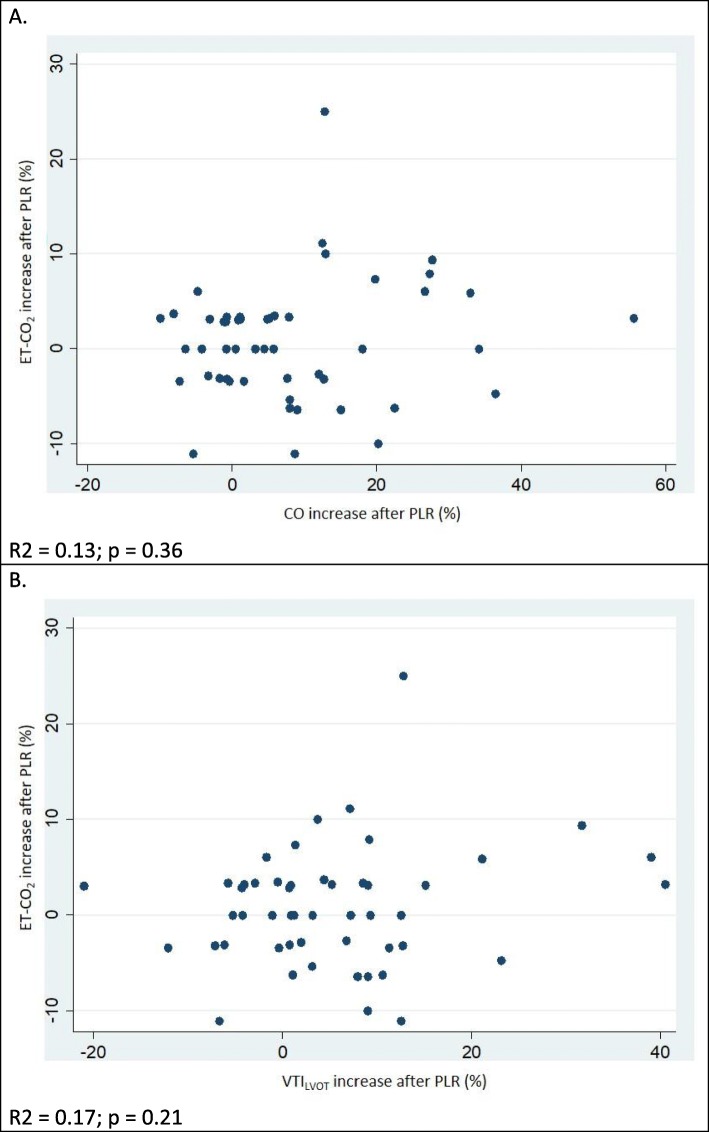
Correlation between PLR-induced changes in ET-CO_2_ and **a** CO and **b** VTI_LVOT_. ET-CO2, end-tidal carbon dioxide; VTI_LVOT_, left ventricle outflow tract velocity time integral; CO, cardiac output

Within the entire population, the PLR-induced percentage change in CO was not correlated with changes in ET-CO_2_ (*R*^2^ = 0.13; *p* = 0.36) (Fig. 4a). There was also no correlation between PLR-induced percentage changes in VTI_LVOT_ and ET-CO_2_ (*R*^2^ = 0.18; *p* = 0.21) (Fig. 4b). Correlation between absolute values of CO and ET-CO_2_ at each measurement point (basal, 90 s, 5 min, 10 min) were also non-significant (*R*^2^ = 0.03, *p* = 0.82; *R*^2^ = 0.18, *p* = 0.20; *R*^2^ = 0.11, *p* = 0.43; *R*^2^ = 0.10, *p* = 0.48, respectively) (Additional file 1: Figure S1).

**Fig. 5:**
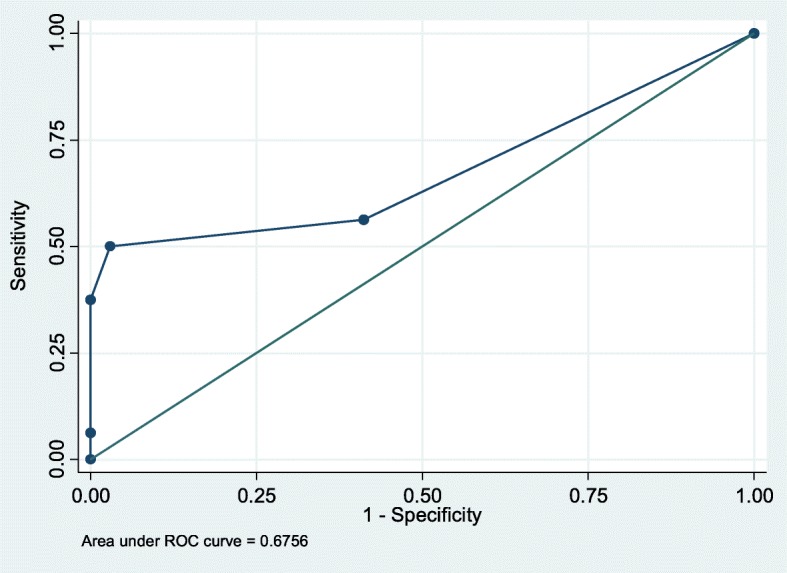
Receiver operating characteristics curves regarding the ability of ET-CO_2_ to discriminate responders (CO increase ≥ 12%) and non-responders after a PLR maneuver. ET-CO2, end-tidal carbon dioxide; CO, cardiac output; PLR, passive leg rise

The area under the ROC curve for ΔET-CO_2_ was of 0.67 ± 0.09 (95% CI 0.498–0.853) (Fig. 5). A ΔET-CO_2_ ≥ 2 mmHg had a sensitivity of 50%, specificity of 97.06%, LR + of 17.00, and LR− of 0.51 for the prediction of fluid responsiveness (Table 4). The PPV for this same cutoff point was 88.9% and the NPV was 80.5%. The performance for the other cutoff points is shown in Table 4. The performance analysis taking ΔVTI_LVOT_ as a gold standard is included in Additional file 1: Table S2.

**Table 4:** Performance of ∆ET-CO2 post-PLR against ∆CO ≥ 12% for the prediction of volume responsiveness

Cut-point (mmHg)	Sensitivity (%)	Specificity (%)	Correctly classified (%)	LR+	LR−
≥ 0	100.00	0.00	32	1.00	NA
≥ 1	56.25	58.82	58	1.37	0.74
≥ 2	50.00	97.06	82	17.00	0.51
≥ 3	37.5	100.00	80	NA	0.62
≥ 6	6.25	100.00	70	NA	0.94
> 6	0.00	100.00	68	NA	1.00

*LR* likelihood ratio, *NA* does not apply

In order not to ignore the probable effect of the heart rate in the determination of the CO, trend curves throughout the PLR maneuver were constructed. In the group of responders, both the VTI_LVOT_ and the HR (baseline 74 ± 11.92, 90 s 80 ± 12.45, *p* = 0.0033) significantly increased from baseline to 90 s. In the group of non-responders, although there was a slight but not significant rise in VTI_LVOT_, HR remained unchanged (baseline 73 ± 13.01, 90 s 73 ± 13.65, *p* = 0.848) (Table 3).

## Discussion

According to the results of this study, the PLR-induced change in CO was not correlated with changes in ET-CO_2_ (*R*^2^ = 0.13; *p* = 0.36) in spontaneously breathing healthy adults. The area under the ROC curve for ΔET-CO_2_ showed little utility of this measurement to discriminate responders from non-responders (0.67 ± 0.09; 95% CI 0.498–0.853) (Fig. 5). Interestingly, a ΔET-CO_2_ ≥ 2 mmHg had a specificity of 97.06%, LR+ of 17.00, PPV of 88.9%, and NPV of 80.5% for the prediction of fluid responsiveness (Table 4). This is consistent with the findings of Monge et al., who found that in mechanically ventilated patients with acute circulatory failure, an absolute increase in ET-CO_2_ ≥ 2 mmHg during PLR was associated with a positive response to fluid administration in all cases [14].

To our knowledge, there is no study that has attempted to evaluate ΔET-CO_2_ post-PLR to predict volume responsiveness in individuals under spontaneous breathing. In this subgroup, one of the most studied tests for this purpose is the ultrasonographic measurement of CO and its surrogates after a PLR maneuver. PLR provides a rapid and reversible ‘self’ volume challenge [18]. A meta-analysis published in 2010 found that this maneuver has a grouped sensitivity and specificity of 89.4 and 91.4%, respectively. The threshold for the prediction of volume responsiveness varied within studies between 8 and 15% [19]. However, although the heterogeneity according to the meta-analysis was not significant, studies included patients both ventilated and in spontaneous breathing. In addition, some studies used CO measurements as the gold standards, others used cardiac index, SV, or VTI_LVOT_. Some used TTE, others transesophageal echocardiography (TEE), minimally invasive CO monitoring through PiCCO® or Vigileo/FloTrac® systems, or invasive CO monitoring through pulmonary artery catheter.

Two studies performed on patients in spontaneous breathing and with echocardiographic measurements of CO should be highlighted. The first, published by Maizel et al., found that a change in CO > 5% after a PLR discriminates responders from non-responders with a sensitivity of 94%, specificity of 83%, PPV of 83%, and NPV of 94%. In this same study, a change in SV > 8% discriminated responders with a sensitivity of 88%, specificity of 83%, PPV of 82%, and NPV of 88% [5]. The second study published by Lamia et al. found that a change in the VTI_LVOT_ > 12.5% discriminated responders with a sensitivity of 70% and specificity of 100% [4].

When evaluating stroke volume, Biais et al. found that a change in SV > 13% measured by TTE after PLR had a sensitivity of 100% and specificity of 80% to predict volume responsiveness [20]. On their side, Préau et al. found that in patients with severe pancreatitis or sepsis, a change in SV > 10% had a sensitivity, specificity, PPV, and NPV of 86, 90, 86, and 90%, respectively [21].

However, the acoustic window for optimal aortic flow Doppler alignment in critically ill patients and the technical difficulty to keep the same probe angle during the PLR maneuver make this measurement not always feasible in the daily clinical practice.

The results of this study suggest that ΔET-CO_2_ after a PLR maneuver lack utility to predict volume responsiveness among healthy adult individuals. However, some considerations must be taken into account. First, although echocardiography is validated for CO determination, with VTI_LVOT_ as the variable with less interobserver variability, it is not free from limitations due to angulation and beam alignment difficulties [22]. This may explain why ET-CO_2_ has shown better correlation with invasive measurements [12, 13] rather than ultrasonographic estimations of CO [14].

Second, there was a concern about the possible influence of heart rate when defining an individual as volume responder, so trend curves throughout the PLR maneuver were constructed. Within responders, both the VTI_LVOT_ (baseline 17.9 ± 3.01, 90 s 20.4 ± 3.42, *p* = 0.0004) and the heart rate (basal 74 ± 11.92, 90 s 80 ± 12.45; *p* = 0.003) had a statistically significant increase from baseline to 90 s. In contrast, within non-responders, although there was a slight but not significant increase in VTI_LVOT_ (basal 19.2 ± 2.67, 90 s 19.3 ± 2.33, *p* = 0.636), HR remained unchanged (baseline 73 ± 13.01, 90 s 73 ± 13.65, *p* = 0.848) (Table 3).

Finally, despite the fact that the ΔET-CO_2_ had poor correlation with CO, and the ROC suggested limited utility to discriminate responders from non-responders, interestingly a ∆ET-CO_2_ ≥ 2 mmHg had a specificity of 97.06%, LR+ of 17.00, PPV of 88.9%, and NPV of 80.5% for the prediction of fluid responsiveness (Table 4). This may be due to the characteristics of the study population. It is known that healthy adults can respond with an increase in CO or SV after a “hed-down tilt” maneuver [23]. However, Parker et al. described how in the healthy human, at rest and in supine decubitus, the ventricular function curve is at its maximum with an end diastolic pressure of the left ventricle of approximately 10 mmHg. Below this point, there is a strong direct relationship between filling pressure and cardiac performance, while with higher filling pressures a plateau occurs. Therefore, in the supine position, the normal heart is usually not in the steep part of the ventricular function curve, but is in a unique position in which the cardiac output is possibly controlled by factors other than the filling pressures of the heart [24]. Probably, in acute and critical illness, there is greater variability in the individual positions among the ventricular function curve, and there is therefore greater probability to induce a change in CO after a volume challenge, as has already been described in mechanically ventilated patients with acute circulatory failure [14].

This study has some limitations that have to be accounted. First, because of the inherent limitations of echocardiography due to angulation and beam alignment difficulties [22], ET-CO_2_ has shown better correlation with invasive measurements [12, 13] rather than ultrasonographic estimations of CO [14]. However, recent studies have demonstrated a significant correlation (*r* = 0.95; *p* < 0.0001) between TTE and pulmonary artery catheter (PAC) CO measurement, with a median bias of 0.2 L/min, limits of agreement between − 1.3 and 1.8 L/min and a precision of 9% for TTE (vs 8% for PAC) [25]. This way we felt confident to avoid an invasive method for the estimation CO in healthy volunteers.

Second, during spontaneous breathing, measurement of ET-CO_2_ by direct or lateral capnography is limited by the inevitable air leak from the system, and the technique has low sensitivity to detect hypoventilation in sedated patients [26]. However, in patients who are not under the effects of sedation, direct or lateral capnographs coupled to nasal cannula have good diagnostic performance [27–29], which may even be comparable with capnography in patients on mechanical ventilation [30].

Third, for the echocardiographic estimation of CO there is an important difficulty when trying to keep the same angle between the probe and the LVOT in both semi recumbent and leg raised positions. For the purpose of this study, we did not opt for the mathematical correction of the effects of angulation, but tried to optimize the angulation of the transducer to make it as parallel to the flow as possible. Finally, the technical difficulties of performing these measurements in individuals with inadequate acoustic windows cannot be ignored, although it was not necessary to exclude any participant for this reason. All ultrasonographic measurements were made by experienced sonographers (VZ, LB, CV).

## Conclusions

According to the results of this study, the performance of ΔET-CO_2_ for the prediction of volume responsiveness in spontaneously breathing healthy adults revealed a sensitivity of 50%, specificity of 97.06%, LR + of 17.00, LR− of 0.51, PPV of 88.9%, and NPV of 80.5% for a ΔET-CO_2_ ≥ 2 mmHg. The area under the ROC curve for ΔET-CO_2_ was of 0.67 ± 0.09 (95% CI 0.48–0.85), suggesting limited utility of this measurement to discriminate responders from non-responders. PLR-induced changes in CO were not correlated with changes in ET-CO_2_ (*R*^2^ = 0.13; *p* = 0.36) in spontaneously breathing healthy adults.

## Additional file

Additional file 1: **Table S1.** Hemodynamic variables and source of origin. **Table S2.** Performance of ∆ET-CO2 post-PLR against ∆VTI ≥ 12.5% for the prediction of volume responsiveness. **Figure S1.** Correlation between absolute values of ET-CO2 and CO at (A) baseline (B) 90 s (C) 5 min and (D) 10 min after a PLR maneuver. (DOCX 177 kb)

## Abbreviations

CO: Cardiac output
CVP: Central venous pressure
ET-CO_2_: End-tidal CO_2_
IVC: Inferior vena cava
PLR: Passive leg rise
SV: Stroke volume
TEE: Transesophageal echocardiography
TTE: Transthoracic echocardiography
VTI_LVOT_: Left ventricle outflow tract velocity time integral
ΔET-CO_2_: End-tidal CO_2_ gradient
