# Supplementary material for: Evaluation of end-tidal carbon dioxide gradient as a predictor of volume responsiveness in spontaneously breathing healthy adults
Source: Intensive Care Med Exp. 2018 Jul 30;6:21. doi: 10.1186/s40635-018-0187-0 (PMC6066588; doi:10.1186/s40635-018-0187-0)
Supplement: Supplementary file 1 — Table S1. Hemodynamic variables and source of origin. Table S2. Performance of ∆ET-CO2 post-PLR against ∆VTI ≥ 12.5% for the prediction of volume responsiveness. Figure S1. Correlation between absolute values of ET-CO2 and CO at (A) baseline (B) 90 s (C) 5 min and (D) 10 min after a PLR maneuver. (DOCX 177 kb) [file 40635_2018_187_MOESM1_ESM.docx]

Additional file 1

| Table S1. Hemodynamic variables and source of origin | |
| --- | --- |
| Heart Rate (HR) | Visoscope |
| Noninvasive Systolic Blood Pressure | Oscillometric tensiometer |
| Noninvasive Diastolic Blood Pressure | Oscillometric tensiometer |
| Noninvasive Mean Arterial Pressure | Oscillometric tensiometer |
| Peripheric O2 saturation (SpO2) | Pulsoximeter |
| Respiratory Rate (RR) | Pulsoximeter |
| End-Tidal Carbon Dioxide (ET-CO2) | TG-920P Sensor |
| Left Ventricle Outflow Tract Velocity Time Integral (VTI_LVOT_) | Transthoracic echocardiography, apical 5 chambers view |
| Left Ventricle Outflow Tract Area (LVOTA) | Transthoracic echocardiography, parasternal long axis view |
| Stroke Volume (SV) | VTIAo x LVOTA |
| Stroke Volume Index (SVI) | SV / Body Surface Area |
| Cardiac Output (CO) | SV x HR |
| Cardiac Index (CI) | SVI x HR |

| Table S2. Performance of ∆ET-CO2 post PLR against ∆VTI ≥ 12.5% for the prediction of volume responsiveness | | | | | |
| --- | --- | --- | --- | --- | --- |
| Cut-point | Sensitivity (%) | Specificity (%) | Correctly Classified (%) | LR+ | LR- |
| ≥ 0 mmHg | 100.00 | 0.00 | 20 | 1.00 | NA |
| ≥ 1 mmHg | 60.00 | 57.50 | 58 | 1.41 | 0.69 |
| ≥ 2 mmHg | 40.00 | 87.50 | 78 | 3.20 | 0.68 |
| ≥ 3 mmHg | 20.00 | 90.00 | 76 | 2.00 | 0.88 |
| ≥ 6 mmHg | 10.00 | 100.00 | 82 | NA | 0.90 |
| > 6 mmHg | 0.00 | 100.00 | 80 | NA | 1.00 |
| LR: Likelihood Ratio; NA: does not apply. | | | | | |

| Figure S1. Correlation between absolute values of ET-CO_2_ and CO at (A) baseline (B) 90 secs (C) 5 mins and (D) 10 mins after a PLR maneuver. | |
| --- | --- |
| A.  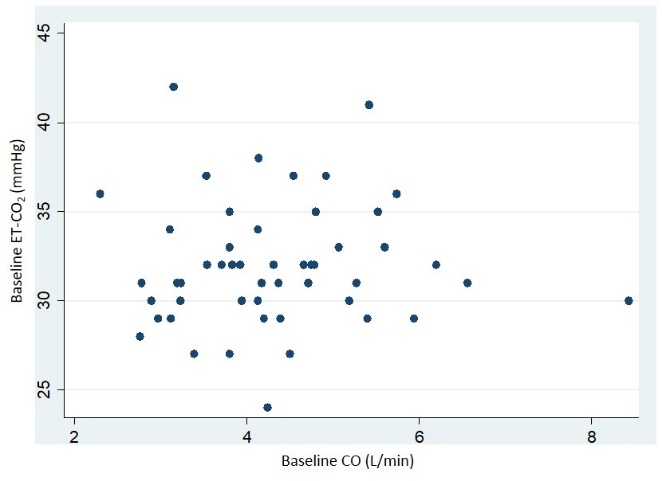  R2 = 0.03; p = 0.82 | B.  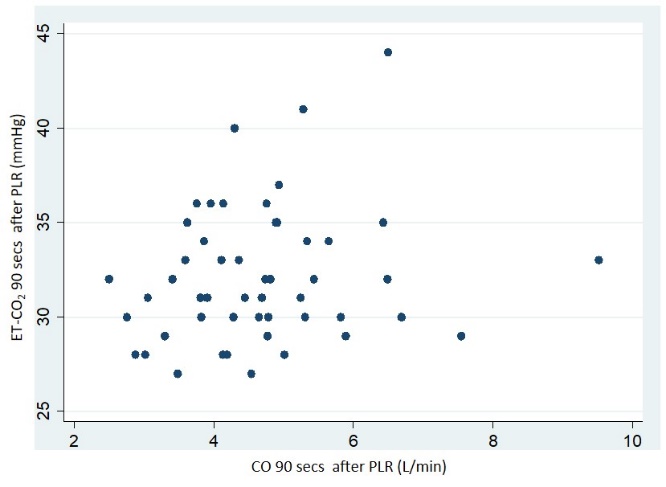  R2 = 0.18; p = 0.20 |
| C.  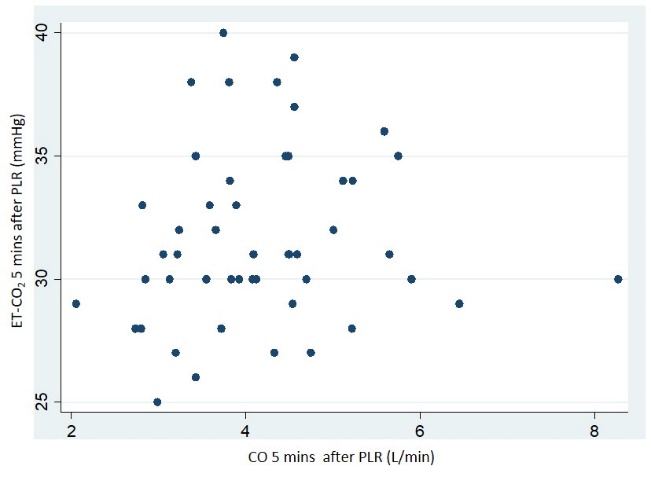  R2 = 0.11; p = 0.43 | D.  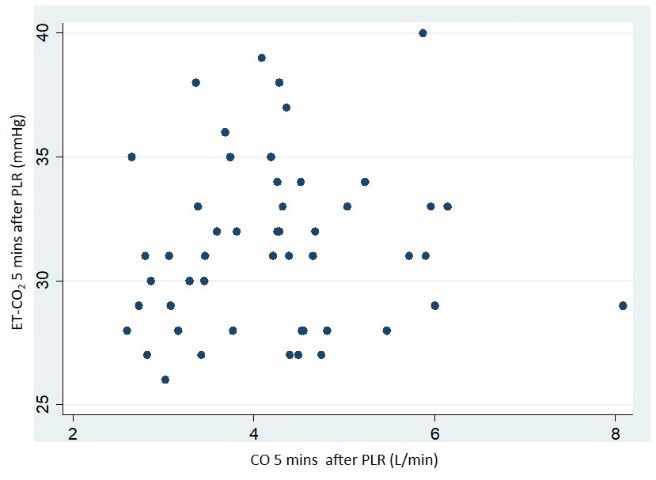  R2 = 0.10; p = 0.48 |
| ET-CO2: end-tidal carbon dioxide; CO: cardiac output; PLR: Passive Leg Rise. | |
